# Integrating family planning into postpartum care through modern quality improvement: experience from Afghanistan

**DOI:** 10.9745/GHSP-D-13-00166

**Published:** 2014-04-15

**Authors:** Youssef Tawfik, Mirwais Rahimzai, Malalah Ahmadzai, Phyllis Annie Clark, Evelyn Kamgang

**Affiliations:** a University Research Co., LLC, Bethesda, MD, USA. Now with Jhpiego, Dhaka, Bangladesh; b University Research Co., LLC, Kabul, Afghanistan. Now in Delhi, India; c University Research Co., LLC, Kabul, Afghanistan. Now with the Ministry of Public Health [Afghanistan], Kabul, Afghanistan; d University Research Co., LLC, Bethesda, MD, USA. Now in Quepos, Costa Rica; e University Research Co., LLC, Bethesda, MD, USA

## Abstract

To address low contraceptive use in Afghanistan, we supported 2 large public maternity hospitals and 3 private hospitals in Kabul to use modern quality improvement (QI) methods to integrate family planning into postpartum care. In 2012, QI teams comprising hospital staff applied root cause analysis to identify barriers to integrated postpartum family planning (PPFP) services and to develop solutions for how to integrate services. Changes made to service provision to address identified barriers included creating a private counseling space near the postpartum ward, providing PPFP counseling training and job aids to staff, and involving husbands and mothers-in-law in counseling in person or via mobile phones. After 10 months, the proportion of postpartum women who received family planning counseling before discharge in the 5 hospitals increased from 36% to 55%, and the proportion of women who received family planning counseling with their husbands rose from 18% to 90%. In addition, the proportion of postpartum women who agreed to use family planning and left the hospital with their preferred method increased from 12% to 95%. Follow-up telephone surveys with a random sample of women who had received PPFP services in the 2 public hospitals and a control group of postpartum women who had received routine hospital services found significant differences in the proportion of women with self-reported pregnancies: 3% vs. 15%, respectively, 6 months after discharge; 6% vs. 22% at 12 months; and 14% vs. 35% at 18 months (*P* < .001). Applying QI methods helped providers recognize and overcome barriers to integration of family planning and postpartum services by testing changes they deemed feasible.

## Abstract (precis)

Modern quality improvement approaches enabled hospital staff to analyze barriers and identify solutions for “how” to integrate family planning into postpartum care. Private spaces for postpartum family planning (PPFP) counseling, along with involving husbands and mothers-in-law in counseling, substantially increased the percentage of women receiving PPFP counseling and their preferred method before discharge. Self-reported pregnancy was also significantly lower up to 18 months post-discharge compared with women receiving routine services.

## INTRODUCTION

Afghanistan has one of the highest maternal mortality rates in the world at 327 deaths per 100,000 live births.^1^ Neonatal mortality is also high at 40 deaths per 1,000 live births, and only 20% of married women use modern contraceptive methods.^1^

Multiple factors contribute to such poor health indicators, including poor health infrastructure, inadequate access to skilled service providers, insufficient access to emergency obstetric and newborn care services, and poor quality of health care.

The value of integrating health services has been recognized universally. Integrating services eliminates missed opportunities for efficiently reaching vulnerable populations with essential preventive and curative services. However, an important challenge in limited-resource settings lies in identifying “how” to integrate services in the face of resource constraints and an overburdened health work force.

The World Health Organization (WHO) defines quality of health care as the:

*proper performance (according to standards) of interventions that are known to be safe, that are affordable by the society in question, and that have the ability to produce an impact on mortality, morbidity, disability, and malnutrition.*^2^^,^^3^

Building on WHO's definition of quality health care, quality improvement (QI) has been defined as:

*a cyclical process of measuring a performance gap; understanding the causes of the gap; testing, planning, and implementing interventions to close the gap; studying the effects of the interventions; and planning additional corrective actions in response.*^4^

The main implication of this definition is that strategies for QI are not “fixed.” On the contrary, QI is a continuous and dynamic process that measures and responds to the results of interventions.

The traditional approach to improving health care quality has been to apply evidence-based guidelines; conduct training; introduce job aids, materials, and equipment; improve supervision; and instill regulation, such as licensing and accreditation. But the new paradigm provided by modern QI, which is derived from 20 years of experience, is based on the understanding that a *system is designed to produce the results it produces*; in order to obtain better results, the system must change.^5^

Modern quality improvement strategies are based on the understanding that systems must change in order to produce better results.

Hence, the emphasis is on analyzing the systems and processes of delivering services and testing changes to obtain better results. This requires thorough analysis of existing procedures and the service delivery workflow to identify areas of potential problems or delays—areas in which change can result in improvement. Resolution of unclear, redundant, or incomplete processes within a broader context is more practical and palatable than placing blame on individuals or on the lack of resources.

The modern paradigm for QI also puts the emphasis squarely on the client in contrast to the traditional medical model that emphasizes the disease. A client-centered perspective draws attention to his/her needs and expectations and frames them within his/her community—not within a health facility. Teamwork is another basic tenet of modern QI. Team members bring valuable insights, not only in identifying and prioritizing problems but also in developing innovative solutions.^5^

The last decade has seen further adaptation of established QI methods to apply evidence-based standards for rapid change and large-scale impact: *collaborative improvement*. Collaborative improvement is a collective improvement activity that unites the efforts of a number of teams to work together to rapidly achieve significant improvements in processes, quality, and efficiency of a specific area of care, with intention of spreading these methods to other sites. Collaborative improvement uses structured, shared learning among participating teams to promote rapid dissemination of successful practices.^6^

This article describes the value of applying modern QI methods to improve service quality and to facilitate the integration of health services in a resource-constrained setting. It discusses how applying such methods can help health care providers analyze the existing service delivery pattern, recognize barriers to integration, and test changes to achieve the desired service integration.

## QUALITY IMPROVEMENT PROCESS

In 2012, we applied modern collaborative QI methods to facilitate the integration of family planning and postpartum care services in 5 high-volume delivery hospitals in Kabul, Afghanistan.

Two of the hospitals were large public hospitals (Malalai and Isteqlal), and 3 were private hospitals (Afghan, Mihdi, and Shinozada). The 5 hospitals combined register approximately 47,000 deliveries annually.

To start the QI process, we first provided an orientation to the leadership and management staff of the hospitals and came to agreement with them on the program's aim and methods. The concepts of modern QI and the implementation steps were explained to the staff.

Accordingly, the hospitals' leadership selected a QI team for each hospital to implement the program. QI teams were comprised of physicians, nurses, and midwives working in the maternity wards with involvement of selected staff working in the family planning units. These QI teams received training on QI concepts and data collection and analysis. They were the leading implementers of the QI process and for measuring its results.

QI teams applied **root cause analysis**, a method of listing the main direct causes for not providing family planning counseling and services to postpartum women in their respective maternity wards and of identifying the reasons for such causes. Hospital staff involved in postpartum care, including doctors, nurses, and midwives, grouped the main root causes of not offering family planning counseling routinely within postpartum care services into the following categories:

- Job expectations
- Skills and knowledge
- Logistics
- Service delivery environment
- Tools
- Culture

QI teams used root cause analysis to identify barriers to integrating services.

Under each category, staff wrote down the most important/direct cause. The outcome of the analysis contributed to directing the participants to the main areas where interventions were likely to result in the desired quality improvement. Subsequently, the teams suggested changes to the process of providing services to make family planning counseling and service provision an integral part of services offered to postpartum women before leaving the hospital.

To capture the most innovative and effective suggestions, the teams applied the process of **brainstorming**, in which each team member gets a chance to suggest interventions/changes to the process of service provision that, from his/her point of view, can contribute most to achieving the desired integration of family planning counseling and services within postpartum care. All suggestions were written on a flip chart. After all QI team members provided their suggestions, the teams discussed the potential benefit and feasibility of each suggestion and selected a group of interventions, or a **“change package,”** that was expected to yield the desired results and that was within the capacity of the hospital to implement.

The QI teams selected a “change package,” or group of interventions they deemed feasible to implement.

In addition, the QI teams conducted brief and **efficient assessments** of the family planning counseling capacity of the service providers working at the maternity ward and the availability of family planning methods and services at their respective hospitals.

The QI teams received training on developing and measuring indicators to monitor the effect of the implemented change package, including how to construct **“time series charts”** to track the changes in the selected indicators over time.

Representatives of the QI teams from the 5 hospitals also met every 3 months to share the changes implemented in their respective hospitals, compare results obtained in each hospital, and discuss challenges for integration and their responses to addressing those challenges.

## DATA COLLECTION

Common indicators that were measured in all participating hospitals and graphed on time series charts to measure the impact of the adopted changes on service integration included the following:

- % of postpartum women who received family planning counseling in the maternity ward before discharge
- % of postpartum women who received family planning counseling with their husbands before discharge
- % of postpartum women who agreed to use a modern contraceptive method
- % of postpartum women who left the hospital with their preferred contraceptive method
- % of compliance with postpartum family planning (PPFP) counseling standards

In addition, we conducted a longitudinal study in Isteqlal and Malalai hospitals in order to measure the outcomes of the PPFP collaborative improvement project. We randomly selected 643 women who received PPFP counseling and 681 women from the same 2 hospitals who received routine hospital postpartum care (that is, they did not receive systematic PPFP counseling), to form intervention and control groups, respectively.

Female family planning counselors from the hospital called these women at 3, 6, 12, and 18 months post-discharge to gather data on the return of menstruation and whether the women thought they were pregnant at the time of the call (that is, self-reported suspected or confirmed pregnancy). If the women reported that they were not pregnant, the interviewers asked whether they were using a contraceptive method and, if so, which method. The family planning counselors also used the opportunity of the follow-up calls to answer questions from women in the intervention group about contraceptive methods and to repeat key family planning messages. (Calls to women in the control group did not include such family planning messages.)

The project team compiled and analyzed the data using Excel to perform frequency distribution and tabulation, and compared the results of intervention and control groups at 3, 6, 12, and 18 months of follow up.

## RESULTS

### Root Cause Analysis of Barriers to Integration

**FIGURE 1.**
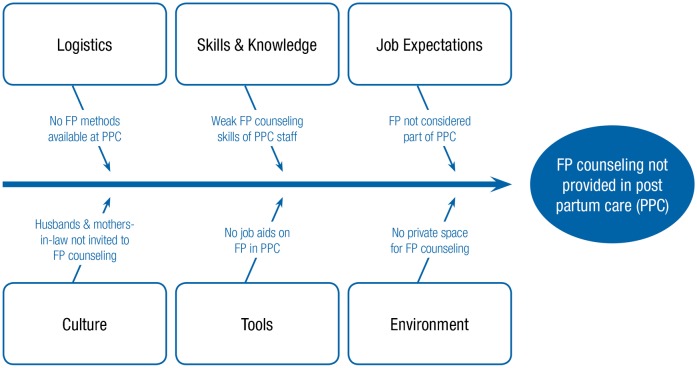
Root Cause Analysis of Barriers to Integrating Family Planning Into Postpartum Care Services Abbreviations: FP, family planning; PPC, postpartum care.

The most important barriers to providing family planning services at the postpartum ward, based on root cause analysis (Figure 1), included:

- Lack of private space for counseling at the postpartum ward
- Inability of some postpartum clients to decide on using contraception without consulting their husbands or mothers-in-law
- Limited family planning counseling skills of postpartum nurses and midwives
- Insufficient access to a variety of contraceptive methods at the postpartum ward

In addition, the assessment of the availability of family planning services present at the hospitals revealed that Malalai and Isteqlal hospitals (large public hospitals) have a partnership with the Afghan Family Planning Guidance Association (AFGA), a nonprofit organization specialized in providing family planning services. Due to this partnership, the 2 public hospitals had adequate family planning services with access to different contraceptive methods, including barrier methods, hormonal contraception, and intrauterine devices (IUDs). However, the smaller private hospitals (Afghan, Mihdi, and Shinozada) had limited family planning services, offering mainly barrier methods and some hormonal contraceptives.

### Change Package of Interventions to Improve Integration

The QI teams introduced innovative changes to address these barriers, including creating a private space for providing family planning counseling near the postpartum ward, providing family planning counseling training to selected postpartum ward staff, providing staff with job aids, and involving husbands or mothers-in-law in the counseling session, if needed, sometimes via mobile phones for those unable to attend in person.

Creating private counseling spaces was particularly important since this made it possible for husbands—who were previously not allowed into the maternity wards—to join their wives for the counseling session. The 2 public hospitals assumed responsibility for building a free-standing counseling room near the postpartum ward and forged links between the postpartum ward and AFGA to provide PPFP services. After receiving PPFP counseling, women in these hospitals who chose a method, such as the IUD, were referred to the AFGA unit located on the hospital grounds near the postpartum ward and the private counseling room.

**Figure:**
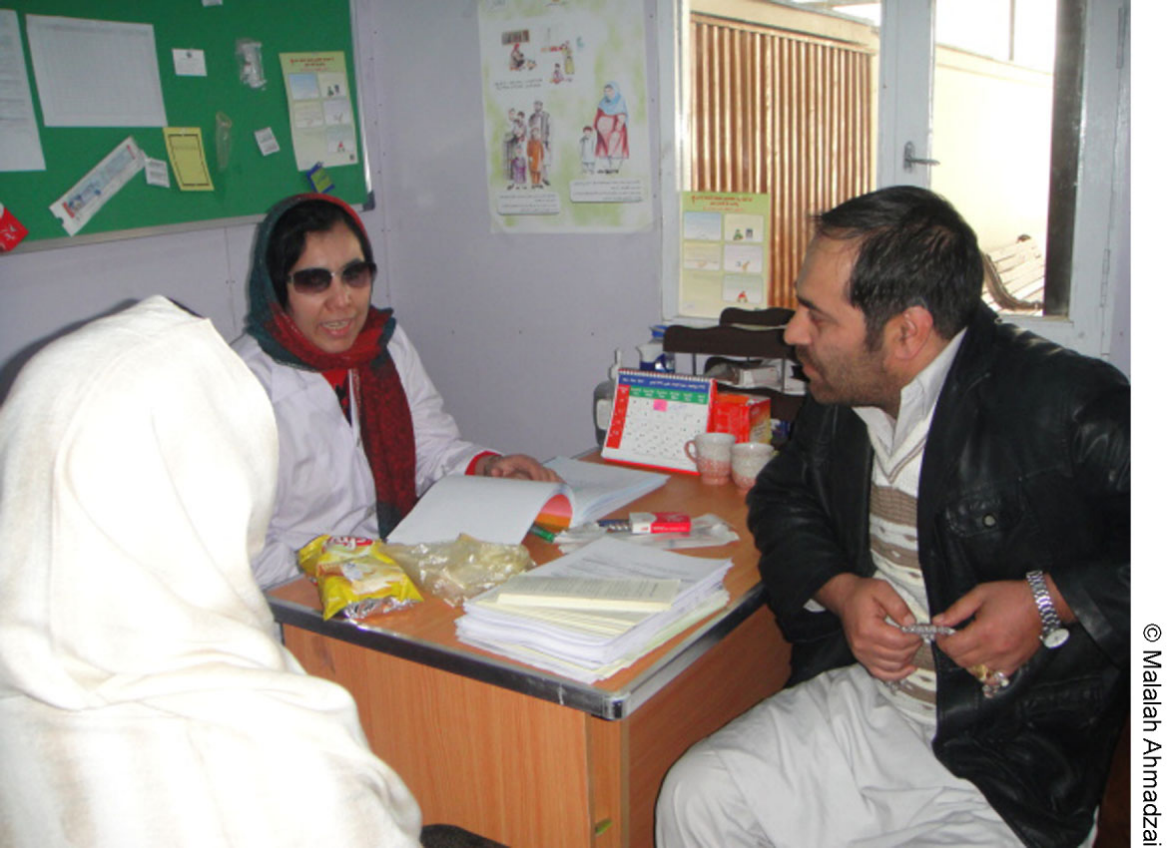
A provider counsels a couple about postpartum family planning in a private space created in Malalai Hospital in Kabul, Afghanistan.

Husbands, who were previously not allowed on maternity wards, were able to join their wives in the newly formed private counseling spaces.

The 3 private hospitals created a private family planning counseling space by repurposing unused rooms close to the postpartum ward. Postpartum women who chose a method not available at the private hospital were referred to family planning providers outside the hospital.

### Effects on PPFP Counseling and Use

Using the time series charts, the QI teams tracked substantial increases in the percentage of postpartum women who received family planning counseling before leaving the hospital, from 36% in January 2012 before the new interventions had been implemented to 55% by November 2012 (among all 5 hospitals combined). In addition, the proportion of women who received family planning counseling with their husbands, either in person or by mobile phone, increased in the 5 hospitals, from 18% in January 2012 to 90% by November 2012.

**FIGURE 2.**
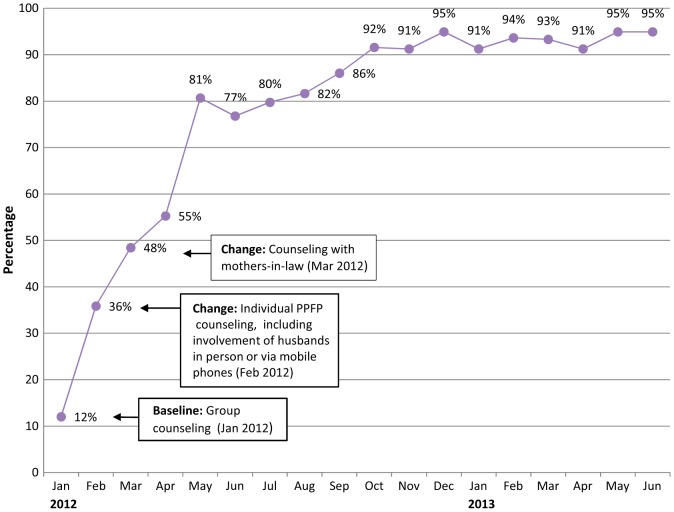
Percentage of Postpartum Women Leaving the Hospital With Preferred Contraceptive Method Among Those Who Received PPFP Counseling, 5 Hospitals, Kabul, Afghanistan, January 2012–June 2013 Abbreviations: PPFP, postpartum family planning.

There was also noteworthy improvement in the proportion of postpartum women who received family planning counseling and left the hospital with their preferred contraceptive method (Figure 2). At baseline, only 12% of postpartum women who received family planning counseling obtained their preferred method. Once systematic PPFP counseling was introduced with women and their husbands, the percentage increased to 36%, and when mothers-in-law were added, to 55%. By the end of the project in June 2013, 95% of postpartum women receiving family planning counseling at the hospital left the hospital with their preferred method.

After family planning was integrated into postpartum services, more women left the hospital with their preferred method.

**FIGURE 3.**
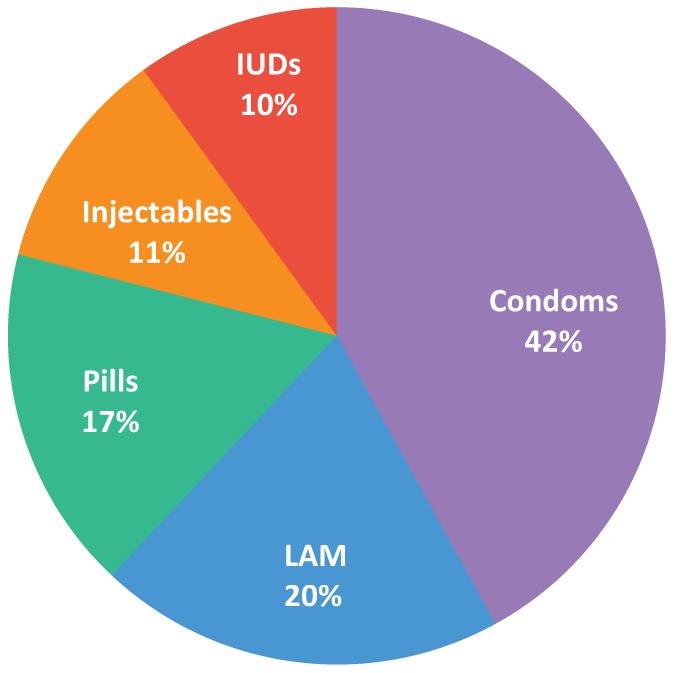
Method Mix Among Postpartum Women,a Isteqlal and Malalai Public Hospitals, Kabul, Afghanistan (N = 580) Abbreviations: IUDs, intrauterine devices; LAM, Lactational Amenorrhea Method. ^a^Among postpartum women who received family planning counseling and decided to use a method.

Among the 580 postpartum women in Isteqlal and Malalai public hospitals who received family planning counseling and decided to use family planning, most chose condoms (42%) or the Lactational Amenorrhea Method (20%). At least 10% chose oral contraceptive pills, hormonal injectables, or IUDs (Figure 3).

Postpartum women in the intervention group at Isteqlal and Malalai public hospitals were significantly less likely than women in the control groups to be pregnant at 6, 12, and 18 months post-discharge (Table). At 18 months, 14% of women receiving systematic PPFP counseling reported they were pregnant compared with 35% of women receiving routine hospital care in the control group.

**TABLE.** Self-Reported Pregnancy (Suspected or Confirmed) Among Intervention and Control Groups, Isteqlal and Malalai Public Hospitals, Kabul, Afghanistan

**Duration Since Delivery**	**Intervention Group**			**Control Group**		
	**Selected Sample**	**Sample Successfully Contacted**	**Pregnant, No. (%)**	**Selected Sample**	**Sample Successfully Contacted**	**Pregnant, No. (%)**
3 months	643	303	0 (0)	681	380	10 (2.6)
6 months	303	217	6 (2.8)	380	235	34 (14.5)
12 months	217	207	12 (5.8)	235	196	43 (21.9)
18 months	207	149	21 (14.1)	196	167	58 (34.7)

Intervention group comprised postpartum women in the 2 public hospitals who received systematic postpartum family planning counseling; control group consisted of women from the same hospitals who received routine postpartum care.

Difference between intervention and control groups is statistically significant (*P* < .001) at 6, 12, and 18 months follow up.

## DISCUSSION

### Solutions for Integrating Family Planning Into Postpartum Care

This study provides valuable insights on the specific challenges of integrating family planning into postpartum care in Afghanistan. Involving husbands in the family planning counseling process posed a special challenge, highlighting the gender aspects of service integration. In Afghanistan, typically men are not allowed in the labor ward, delivery room, or postpartum ward, which posed a particularly difficult challenge. QI approaches inject a culture of creativity that allows involved staff to “think outside the box” to test innovations that they otherwise would not be allowed to try. In this instance, a private counseling room was created by the hospital itself, and husbands were invited, personally or by mobile telephone, to participate in the counseling session.

The inability of the maternity staff to provide a wide range of family planning services, particularly long-acting methods such as IUDs, posed another important challenge. Providing such clinical contraceptive methods is more difficult than providing certain resupply methods, such as condoms, on postpartum wards. Again, thinking outside the box, the staff realized that long-acting methods were available in the family planning unit of the same hospital, and that improving the link between the maternity ward and the family planning unit could result in expanding contraceptive options for postpartum women. When the postpartum ward was not able to satisfy a particular family planning need, the link created with AFGA maximized family planning options for postpartum women.

Improving links between maternity wards and family planning units of the hospitals improved contraceptive options for postpartum women.

Often, health programs focus on advocating the integration of services, emphasizing the potential health, programmatic, and financial benefits of integration. However, the question of “how” to overcome barriers to service integration is usually not addressed adequately. Health care providers generally appreciate the value of integration. Modern QI offers practical approaches to help providers understand the barriers to integrating services in their own context and to guide them in identifying and testing interventions to overcome such barriers.

### Applying Modern QI: Lessons Learned

This study provides evidence that clinical providers in limited-resource settings are capable of absorbing concepts of modern QI and applying them, with some technical support, to integrate services. QI teams in the selected hospitals received orientation on QI and on tools for analyzing health services processes, such as patient flow analysis and root cause analysis. In addition, the teams received training in data collection, monitoring, and use.

The involvement of hospital leadership in the QI process was essential to gain support for the improvement intervention and to enable the QI teams to apply changes to the current system and test their impact. The service providers involved in the QI process reported that the approach was easy to grasp and could be used to improve other health services. They felt encouraged and empowered when they were able to measure their own results and see an improvement in the quality and volume of services provided. In addition, the process augmented the sense of teamwork and cooperation between different staff categories because it required involvement of doctors, nurses, and midwives working together.

The results show the value of QI approaches in helping health care providers to functionally integrate services, such as integrating family planning into postpartum care services. The study adds to the growing body of evidence that modern QI methods can achieve significant improvements in quality of care, even in underdeveloped health systems such as in Afghanistan. The same approach of developing staff capacity to analyze their own system, identify challenges for integration, and select and test changes to achieve integration has potential for application to integrating a wide range of services.

Our experience of applying modern QI approaches at the hospital level in Kabul provides several lessons for Afghanistan and beyond. First, involving and gaining the support of the institutions' leadership is essential. To apply QI effectively, staff members need a non-threatening environment where they feel free to contribute their ideas openly in order to expose problems and suggest solutions. Such an enabling environment can be established only through the support of the institution's leadership.

Obtaining support from institutional leadership is essential to ensuring the success of modern QI approaches.

Second, service providers and other staff who are intimately involved in the services must be involved in the QI process. Such individuals tend to have first-hand knowledge of the barriers to quality service and hence are able to come up with creative and practical solutions to overcome the identified barriers.

Staff with first-hand knowledge of service barriers should be involved in the QI process.

Third, the QI process should be kept simple enough so that service providers own it. Simplifying the process increases the likelihood that service providers can establish and measure indicators to monitor the progress of the QI project. It also helps ensure that they can use results from monitoring activities to take corrective actions to improve the QI process and the services delivery outcome.

### Limitations

The methodology used to track women after their hospital stay resulted in a potentially biased sample and in smaller sample sizes over time. The initial sample at 3 months post-discharge was randomly derived, but for each subsequent follow-up period, the sampling frame consisted only of women who had been successfully contacted during the previous follow-up period. In addition, since follow up was conducted over the telephone, the sample may have been biased in favor of more socioeconomically privileged women who had access to telephones. However, almost 90% of women originally sampled in both groups had access to a mobile telephone number. Finally, the self-reported nature of current pregnancy status could also have been biased. However, these sources of potential bias could be expected to affect the control and intervention groups equally.

## CONCLUSION

Integration of health services requires more than simply emphasizing the importance of integration; providing simple approaches and tools to guide health staff to address “how” they can integrate services helps to ensure success. In addition, engaging providers in analyzing their own care processes can stimulate innovative problem-solving and creative solutions. Modern quality improvement approaches have an important role in empowering service providers to identify barriers to health services integration, analyze the causes of the barriers, and test interventions to overcome them.
